# The Effect of Welding Energy on the Microstructural and Mechanical Properties of Ultrasonic-Welded Copper Joints

**DOI:** 10.3390/ma10020193

**Published:** 2017-02-16

**Authors:** Jingwei Yang, Biao Cao, Qinghua Lu

**Affiliations:** 1 School of Mechatronics Engineering, Foshan University, Foshan 528000, China; qhlu@fosu.edu.cn; 2 School of Mechanical and Automotive Engineering, South China University of Technology, Guangzhou 510640, China; mebcao@scut.edu.cn

**Keywords:** ultrasonic welding, copper, plastic deformation, wavy interface

## Abstract

The effects of welding energy on the mechanical and microstructural characteristics of ultrasonic-welded pure copper plates were investigated. Complex dynamic recrystallization and grain growth occurred inside the weld zone during ultrasonic welding. At a low welding energy, a thin band of straight weld interfaces was observed and had an ultra-fine grain structure. With an increase in welding energy, the weld interface progressively changed from flat to sinusoidal, and eventually turned into a convoluted wavy pattern, bearing similarities to shear instabilities, as observed in fluid dynamics. The lap shear load of the joints initially increased and then remained stable as the welding energy increased. The tensile characteristics of the joints significantly depended on the development of plastic deformation at the interface. The influence of the microstructure on the hardness was also discussed.

## 1. Introduction

Copper and corresponding alloys have been widely utilized in battery assemblies in the battery electric vehicles (BEVs) industry, due to their merits, including the high electrical and thermal conductivities of both, and the subsequent favorable combination of strength and ductility [1]. Due to the high power and capacity requirements for BEVs, the lithium-ion battery pack is usually assembled from several hundreds, or even thousands, of battery cells, mainly pre-designated by the cell configuration and pack size of the final product. Therefore, a high demand exists for joining in battery pack manufacturing. However, the joining of copper by traditional fusion welding processes is usually difficult because of high thermal conductivity and serious oxidation at the melting temperature of copper [2,3]. Moreover, a significant amount of heat is generated in the weld, leading to a rapid increment in the cell temperature. This will destroy the separator in the cell, causing an internal failure [4]. Therefore, in order for such problems to be overcome, solid-state welding methods, such as friction stir welding (FSW) and ultrasonic welding (USW), have been regarded as alternative solutions for the joining of copper in battery pack manufacturing.

The FSW constitutes an energy efficient and environmentally friendly joining method, and was invented at The Welding Institute in 1991 [5]. As a relatively new solid-state welding technique, the FSW has proven to be demonstrably better than conventional welding methods for the joining of copper and aluminum alloys, due to lower heat inputs and welding temperatures [6]. Unfortunately, the FSW technique is being faced with challenges in electronic and BEV industries, where a significant amount of joining of miniature work pieces of irregular shape and materials, with various thickness combinations, is required [7].

The USW is another kind of solid-state joining process, which utilizes high frequency ultrasonic vibration to produce joints under a static clamping force. Although USW originated in the 1930s, it was mainly used in the joining of thin wires and foils, due to the USW system power limitations. More recently, as the high power USW system has become more widely available, particularly during the last few years, considerable research has been conducted on the joining of thick gauges with various material combinations. As an example, in similar metal welding, Jahn et al. [8] and Bakavos et al. [9] investigated the effects of welding energy on the evolution of weld microstructures and weld strength in USW. It was reported that the interface changed from a planner to a wavy morphology, and that the weld strength increases as the welding energy increases. Shin and de Leon [10] studied the process parameters, including the vibration amplitude, the welding duration, the horn, and the anvil tip patterns for the A5052 Al alloy joints. It was concluded that the mechanical properties and weld quality of the joints were governed by these parameters. Similar results were reported in ultrasonic-welded Mg alloys [11]. It was noted that the microstructure, crystallographic texture, and mechanical properties of the Mg (AZ31B) joints, were highly affected by the welding energy parameter [12]. The USW is also employed in dissimilar metal joining, such as the Al–Mg, Al–Fe and Al–Cu, in order for sound joints to be obtained. Patel et al. [13] investigated the Al–Mg joints under the effect of welding energy. It was reported that the weld strength and evolution of the IMCs of the Al–Mg joints were highly affected by the welding energy. In addition, interface liquation was observed for longer welding durations at temperatures below the eutectic reaction temperature in the Al–Mg binary system in the USW [14]. The growth rate of the IMC layer in the USW was also observed to be over double the growth rate observed under static conditions [15]. Similar investigations were performed on the Al–Fe joints [16,17] and Al–Cu joints [18]. Despite the ability of dissimilar joints production, the rapid formation of IMCs is still unavoidable in the USW. This consequently affects the mechanical properties of the joints. In order for excellent dissimilar joints to be obtained, certain reports [19,20,21] have suggested that the mechanical properties were highly improved by the diffusion barrier layer between the work pieces. It was concluded that the diffusion barrier layer limits the formation of the IMCs to a high extent, resulting in higher quality welds. Furthermore, extensive research has been conducted on the structural components produced from lightweight alloys in automotive structures, whereas the literature on the USW of thick copper alloy gauges is limited. In addition, since the USW is currently the most utilized joining technique in battery manufacturing, the welding ability of copper alloys by the USW needs to be clarified.

In brief, the objective of this work was to gain a better understanding of the microstructural evolution of thick copper gauges when subjected to high power ultrasonic spot welding. A detailed investigation with various welding energies was executed. In addition, the effect of microstructural evolution on the mechanical properties was also investigated in detail. 

## 2. Materials and Methods

**Figure 1:**
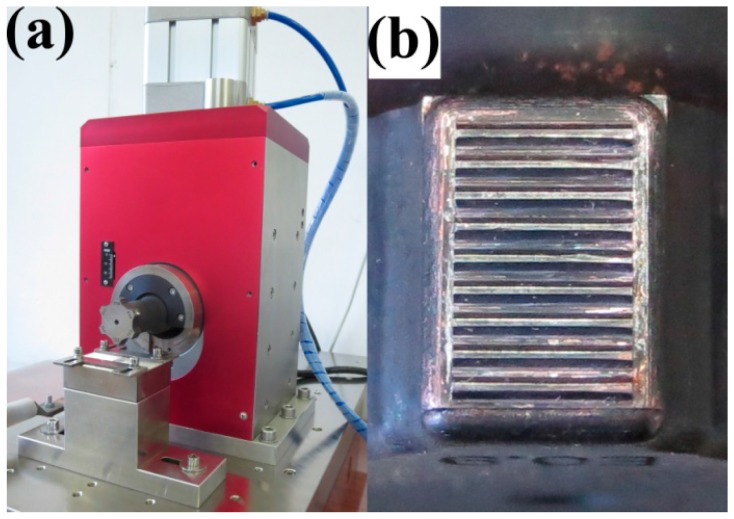
(**a**) USW system employed in the study; (**b**) sonotrode tip.

The base metal used in the present study was a commercially pure copper sheet of a 0.8 mm thickness, with a ½ H condition. The chemical composition (wt %) was of 0.1O-0.01S-0.01As-0.01Pb in stoichiometry. The copper plate was cut down to rectangular weld test specimens of 100 mm in length and 25 mm in width. Additionally, the coupons, without having been cleaned or surface-treated prior to welding, were welded at the center of the 25 mm overlap with a high-powered ultrasonic welding machine. The welding system employed was a lateral drive Telsonic-M5000 machine (Telsonic, Bronschhofen, Switzerland), operating at 20 kHz in resonance frequency, with a 4 kW maximum output power (Figure 1a). The 5 mm × 7 mm sonotrode tip had a serrated surface comprised of nine parallel teeth (Figure 1b). USW was conducted at a constant clamping force of 2350 N, combined with various welding energies of 200, 400, 800, 1200, 1600, 2000, and 2400 J, adjusted from the machine.

The macrostructure of the joins was examined by optical metallurgical microscopy (LEICA-DMI, Wetzlar, Germany). The metallographic samples were cross-sectioned in parallel to the ultrasonic vibration direction, polished, and then etched with a 10 mL solution of distilled water, 1.7 mL hydrochloric acid, 20 g chromium oxide, and 2 g of sodium hyposulfite. The grain structure of the as-polished welds was measured by a scanning electron microscope equipped with an HKL EBSD detector (Oxford Instruments, Oxford, UK). The data processing was performed by the MTEX software (Chemnitz, Germany). Moreover, Vickers hardness tests with a 15 g load were performed for 10 s, across the welded joint. Tensile lap shear tests were executed at room temperature with a constant crosshead speed of 1.0 mm/min by an AGX-50kNXD testing system (Shimadzu, Kyoto, Japan). The relative bond density for the variety of welding energies was estimated from the length of the metallic joining area’s usage, located at the weld interface in the weld zone. The interfacial temperatures were measured by a 0.15 mm diameter k-type thermocouple, implanted just below the top sheet through a precision drilled hole. The temperature variations were recorded by the NI 6133 data acquisition system (National Instruments, Austin, TX, USA).

## 3. Results and Discussion

### 3.1. Macrostructural Investigations

**Figure 2:**
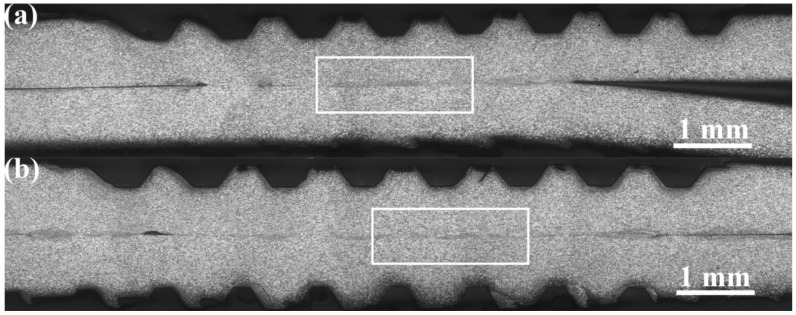

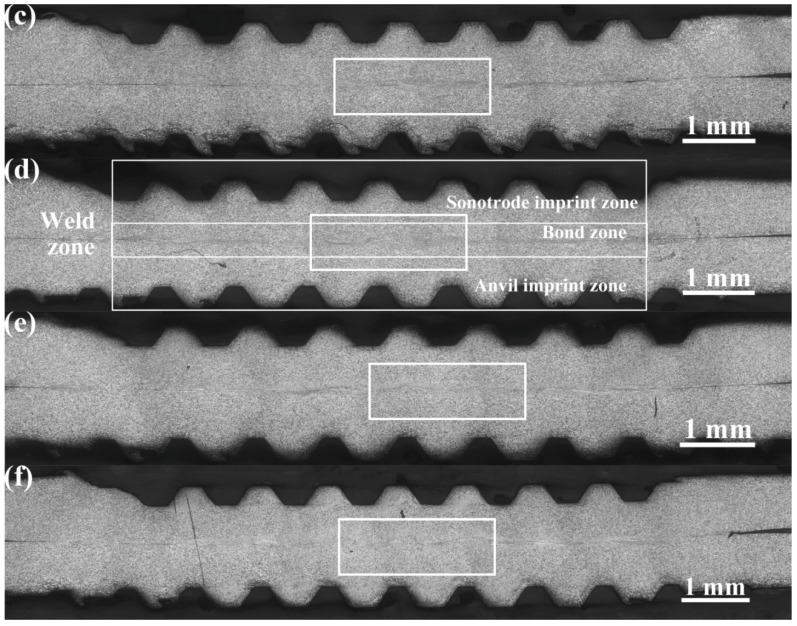
Typical micrographs for macrostructure of cross-sections of USW joints produced from welding energy increase: (**a**) 400 J; (**b**) 800 J; (**c**) 1200 J; (**d**) 1600 J; (**e**) 2000 J and (**f**) 2400 J.

In Figure 2, typical micrographs of the macrostructure for the weld cross-sections of the USW joints, produced with increasing welding energy, are presented. The defect-free joints were obtained from a wide variety of welding energies ranging from 1600 to 2400 J, under a constant clamping force of 2350 N. Based on the macrostructural characteristics, four distinct regions, including the sonotrode imprint zone, the bond zone, the anvil imprints zone, and the base metal exhibited in the USW joints, are presented in Figure 2d.

During low welding energies, the anvil imprints zone was not clearly visible, because the limited input energy was mainly consumed in the sonotrode imprint zone and the bond zone. As a result, the acousto-plastic effect (APE) and the temperature of the lower sample were not sufficiently adequate for the soft metals to be plastically deformed. As the welding energy intensity increased, both the APE and temperature rose, and the ridges of the anvil started to penetrate the sample surface. From Figure 2c,d, it can be observed that the ridges of the anvil reached their full depth at the approximate intensity of 1600 J, whereas an intensity of 1200 J was adequate for the ridges of the sonotrode tip. At a higher energy input, the metal in the weld zone became so soft that it began to extend along the direction of the anvil. This led to a thinning of the weld zone (Figure 2e,f). Therefore, the formation of the USW joints was determined by the amount of the welding energy, simultaneously with the transfer and conversion of ultrasonic energy.

### 3.2. Microstructural Investigations

**Figure 3:**
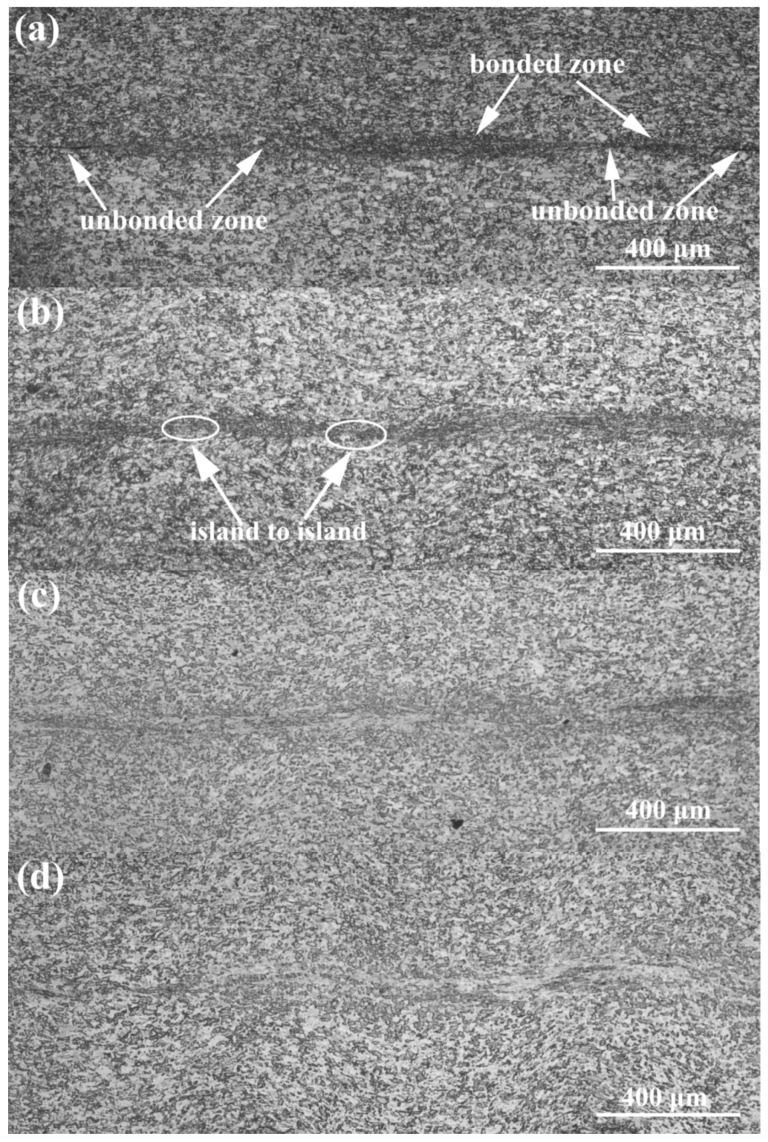

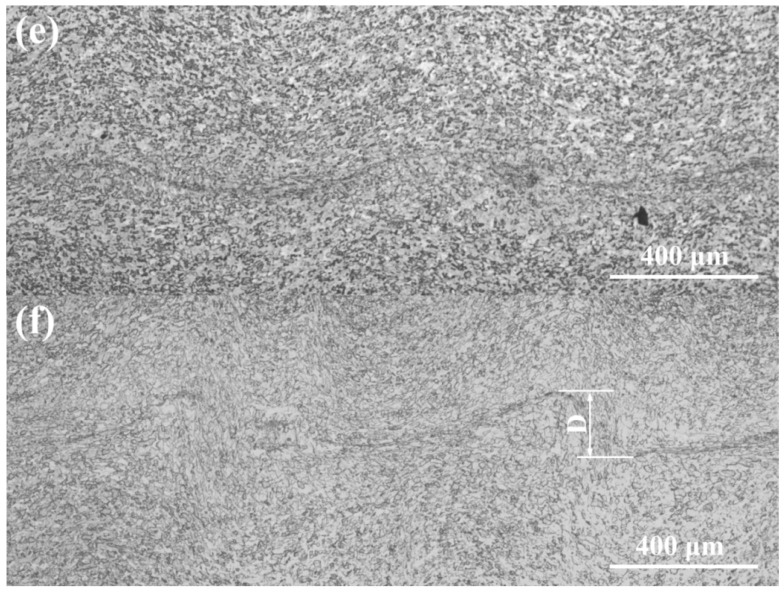
Enlarged regions of the bond zones from the weld cross-sections shown by the white boxes in Figure 1: (**a**) 400 J; (**b**) 800 J; (**c**) 1200 J; (**d**) 1600 J; (**e**) 2000 J and (**f**) 2400 J.

Typical microstructures of the bond zones in relation to the welding energy are presented in Figure 3. From Figure 3, it can be observed that, as the welding energy is increased, it evolves from the localized microwelds to the whole weld zone, and the morphology of the weld interface changes from a straight to a wavy interfacial pattern. During low welding energies, the weld formation developed heterogeneously at the specific regions under the sonotrode tip ridges. This probably resulted from the existence of a higher pressure near the sonotrode tip ridges. A large unbound area was concentrated at the weld line, and the bonding zone appeared as isolated, island-shaped areas in various sizes (Figure 3a). As the welding energy continued to increase, the bonding rapidly expanded along the vibration direction and tended to coalesce into a larger bond. Consequently, the width of the plastic deformation zone was not uniform along the weld interface, and the width between the bonding island-shapes was relatively small (Figure 3b). As the welding energy increased to approximately 1200 J, the increase in the length of the plastically deforming region accelerated and the width became more uniform (Figure 3c). From Figure 3a–c, it can be observed that, for low energies (400–1200 J), plastic deformation was highly localized to the weld line, and the microbonds increased in density spreading and coalescing, as the welding energy increased. Large unbounded areas were still observed on the weld interface. The weld formation at this stage was closely related to the total input energy, the energy transfer process, and the generated heat on the interface. Firstly, the contribution of the relative displacement occurring between the weld coupons, led to the appearance of frictional heat on the weld interface. Due to the short welding duration, the dissipation of the frictional heat was limited. Therefore, the heat was mainly concentrated on the weld interface. Secondly, the total input energy was low. In addition, intense friction and squeezing existed in the sonotrode imprints zone and the anvil imprints zone, that unavoidably consumed a portion of the input energy. As a result, both the plastically deforming and unbounded region were found at the weld line. Therefore, the weld line appeared as a flat, thin band when studied macroscopically.

**Figure 4:**
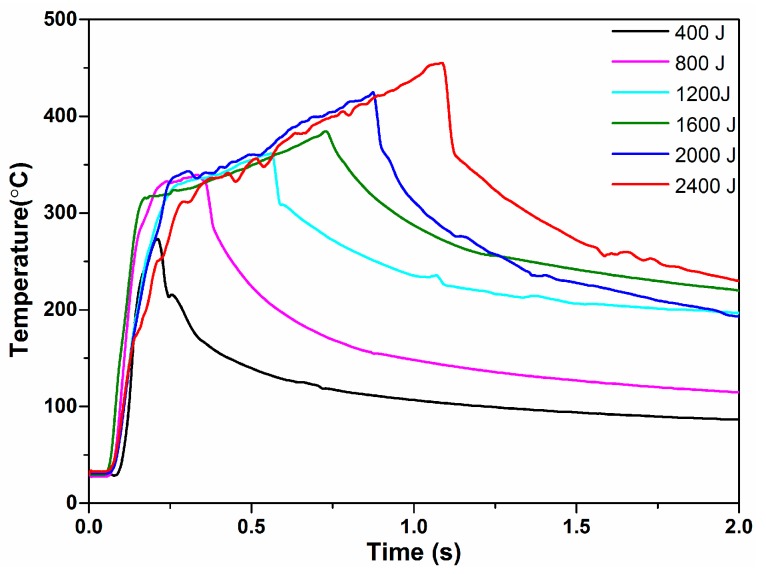
Temperature variation of weld interface recorded during USW for various welding energies.

At high energy values (Figure 3d–f), the plastically deforming zone expanded and the weld interface changed from sinusoidal into a convoluted wavy pattern. The morphology of the weld interface resembled the Kelvin-Helmholtz instability wavy morphology, as a continuum mechanics phenomenon in Newtonian fluid flux, similar to the studies reported by Nassiri et al. [22]. When the welding energy reached 2400 J, the plastic deformation became quite intense and expanded throughout the entire weld zone. Several origins of the various interface morphologies were observed. The most obvious was that most of the energy was consumed in the weld zone during higher energies, sufficiently elevating the temperature near the weld interface (Figure 4) for the metal to become soft enough to plastically deform. In USW, the effective stress distribution inside the metal coupon was dynamically changed by pressure and shear stress, caused by ultrasonic vibration. In fact, the appearance of wavy interfaces in USW could be regarded as a special case of the general phenomenon of wave formation of stress under certain flux circumstances. It is known that reflection and interference exist in stress wave propagation. This results in stress being superposed in the coupons. Therefore, transitory local stress concentration regions appear. When the effective stress of certain areas inside the coupons exceeded the yield stress, plastic deformation occurred. The plastically deformed metal then flowed with the stress wave. However, due to the uneven distribution of the stress, the micro motion inside the plastically deformed metal was different. The speed vector had inconsistencies, easily leading to complex plastic flow. At high energy values, the complex morphologies observed on the interface mainly depended on the amount of shear deformation and plastic strain. The higher the welding energy was, the greater the shear deformation and the more complex the morphology of the interface. At low welding energies, the interfaces were straight, whereas when the welding energy increased, a transition occurred to the wavy interface. It was reported by Bakavos et al. [9] and Haddadi et al. [23], that wavy interfaces were also observed from the ultrasonic-welded 6111Al.

**Figure 5:**
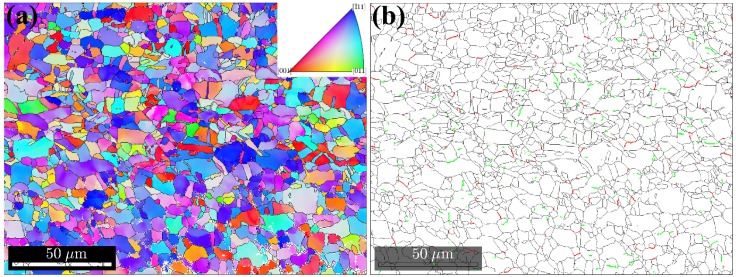

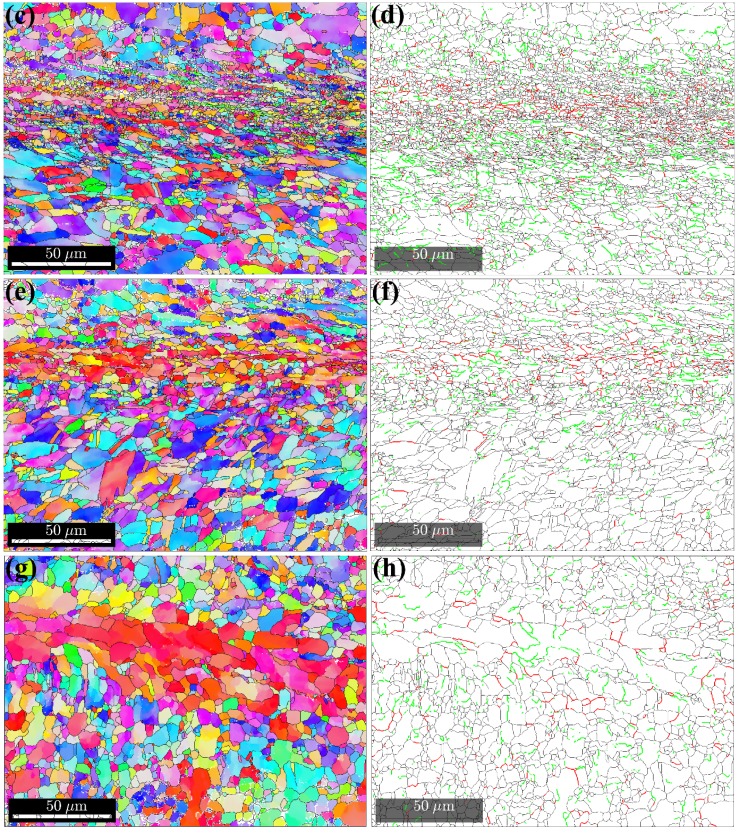
(**a**,**c**,**e**,**g**) EBSD orientation maps and (**b**,**d**,**f**,**h**) grain boundaries maps of (**a**,**b**) base metal and at the center of the weld zone for joints with weld energy of: (**c**,**d**) 800 J; (**e**,**f**) 1600 J and (**g**,**h**) 2400 J.

In Figure 5, the EBSD images of the base metal and the bond zone at the center of the joints, under various welding energies, are presented. The black, red, and green lines represent the high-angle grain boundaries (>15°), the low-angle grain boundaries (<15°), and the subgrain boundaries, respectively. The microstructure of the PM was characterized by the coarse grains with a high number of annealing twins and high-angle grain boundaries, as presented in Figure 5a,b. When the welding energy was below 800 J, the microstructure was essentially dynamically recrystallized. As presented in Figure 5c, the fine equiaxed or elongated new grains were produced at the weld line. The size of the grains in the deformed matrix was uniform. These are typical dynamic recrystallization microstructures, according to [24]. The high density of the low-angle boundaries observed in Figure 5d, especially the formation of the discontinuous subgrain boundaries, was probably evidence for the recovery of dislocations, into the subgrain boundaries [23]. Compared to Figure 5f,h, the density of the subgrain boundaries decreased as the welding energy increased. This suggests that the recovery of dislocations promoted the rotation of sub-grain boundaries, into the high-angle grain boundaries. The subgrain rotation and subsequent rotation recrystallization were probably the main mechanisms for recrystallization. When the input energy reached 1600 J, a non-uniform microstructure with coarse grain zones and fine grain zones was observed in the bond zone (Figure 5e). It was indicated that the grain size of the bond zone was of a lower size than the grain size of the base metal, when the welding energy was below 1600 J. As the welding energy increased to 2400 J, the mean grain size became higher than that of the base metal. The main reason for this was attributed to a large amount of strain and excessive heat which had accumulated at the bond zone, as the welding energy increased. As a result, it caused a serious growth of the recrystallization grains. Moreover, due to the lack of the second phase ions in pure copper, the grain growth could not be inhibited.

### 3.3. Mechanical Properties

**Figure 6:**
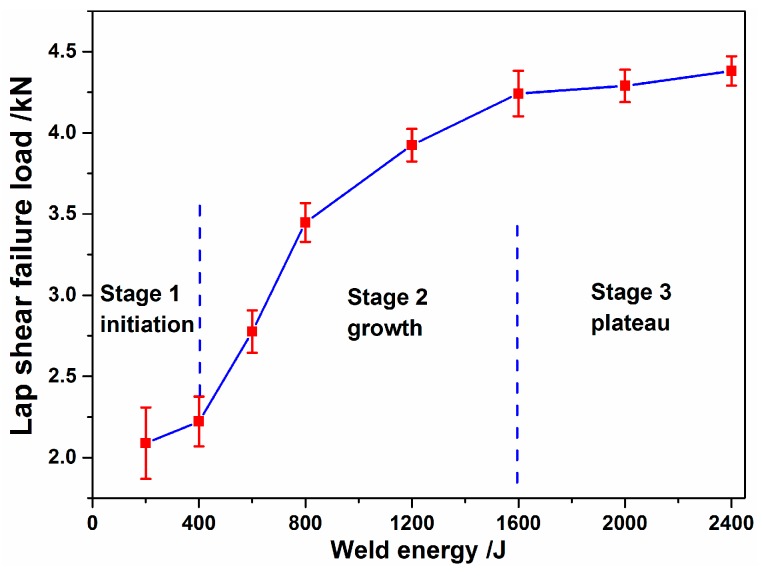
Effect of welding energy on the peak load of as-welded Cu/Cu USW joints.

In Figure 6, the peak lap shear loads measured from the weld joints, having been plotted against the welding energy with a constant clamping force of 2350 N, are presented. It is observed that the load curves can be described in three stages: the initiation stage, the growth stage, and the plateau stage. The peak lap shear loads increased as the welding energy increased from 200 to 1600 J. At higher energies, the plateaus reached a peak load with an approximate upper limit of 3.3 kN.

During the initiation stage, the coupons being welded between the sonotrode and the anvil were brought together by the clamping force. High frequency vibrations from the transducer caused the sonotrode motion to be parallel to the weld surface. Due to the tangential force caused by the sonotrode, a relative motion occurred between the coupons. The surface asperities were plastically deformed and progressively sheared by the friction force between the weld surfaces. The contaminants surrounding the asperities were crushed as a result of contact between the local metals. These areas were referred to as weld islands in Figure 3a. Due to small bonding areas, the peak lap shear loads value was low. As the welding energy increased, more weld islands were formed, and some grew and coalesced, leading to increased joint areas. The weld interface changed from the original plain form, to larger weld island formations, and the welding proceeded to a growth stage. During this stage, because the growing bonding areas induced by the shear stress were mixed, the lap shear failure loads rose rapidly. As the welding energy input increased, the joining between the metal continued, until the bonding areas reached a certain point of growth, being equal to or slightly larger than the corresponding area of the sonotrode tip (Figure 1). At that point, the joint appeared to become saturated and entered the plateau stage, during which the joint strength increased asymptotically and approached a limiting value of approximately 4.3 kN. That was the effect of the increased energy on the weld strength, and was not as significant in the plateau stage. 

**Figure 7:**
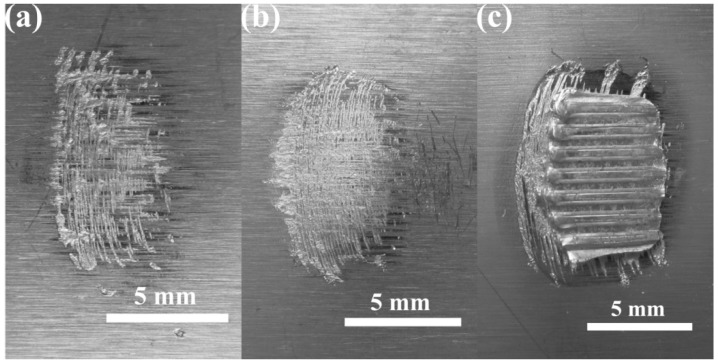
The fractures surfaces of Cu-Cu joints after lap shear testing revealing a change in fracture mode from interfacial cleavage to nugget pull-out: (**a**) 400 J; (**b**) 800 J and (**c**) 2000 J.

Two representative fracture modes, the interfacial cleavage mode and the pull-out mode, were observed in the Cu-Cu joints produced by USW, as presented in Figure 7. Concerning the interfacial cleavage mode, failure occurred on the interface. Concerning the pull-outmode, the weld nugget stuck on the bottom sheet and the fracture path was deployed along the edges of the weld nugget through the top copper sheet. The transition of the failure mode from the interfacial cleavage to the pull-out mode occurred when the welding energy reached 1600 J. This coincided with the welding conditions, leading to the nugget being pulled-out in the plateau stage, as presented in Figure 6. At welding energies below 1600 J, the copper sheets could not be successfully welded together.

**Figure 8:**
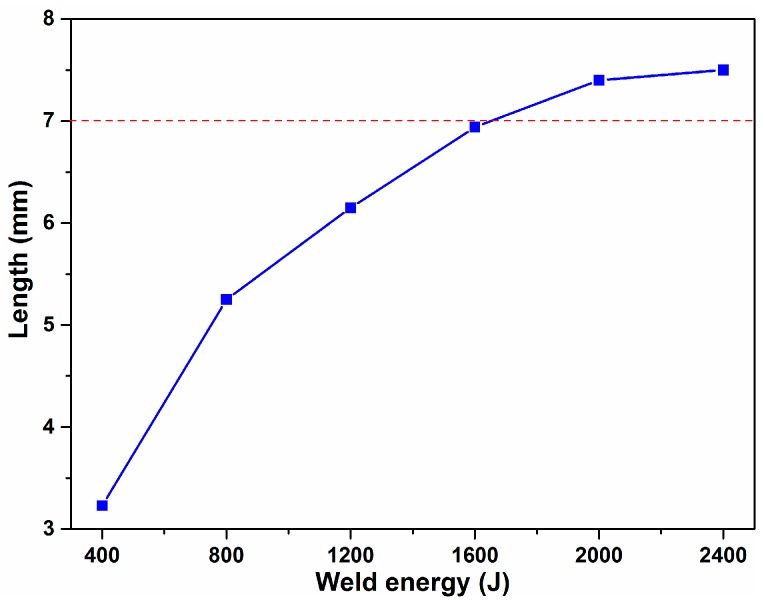
Length of metallic joining in the weld zone as the welding energy increased.

The length of the metallic joining in the weld zone, as a function of welding energy, is presented in Figure 8. An increase of the welding energy was accompanied by an increase in the length of the metallic joining, which was a decisive factor for the interfacial strength. As a result, when the welding energy increased from the as-welded condition, the fracture mode was transferred from the interfacial cleavage, to the nugget pull-out mode. In addition, the length of the metallic joining was slightly larger than the sonotrode (7 mm) length. Due to high temperatures in the welding zone during high energies, the plastic deformation resistance of the material was low, permitting the sonotrode to fully penetrate the workpiece. A new bonded region was formed at the edge of the weld tip.

**Figure 9:**
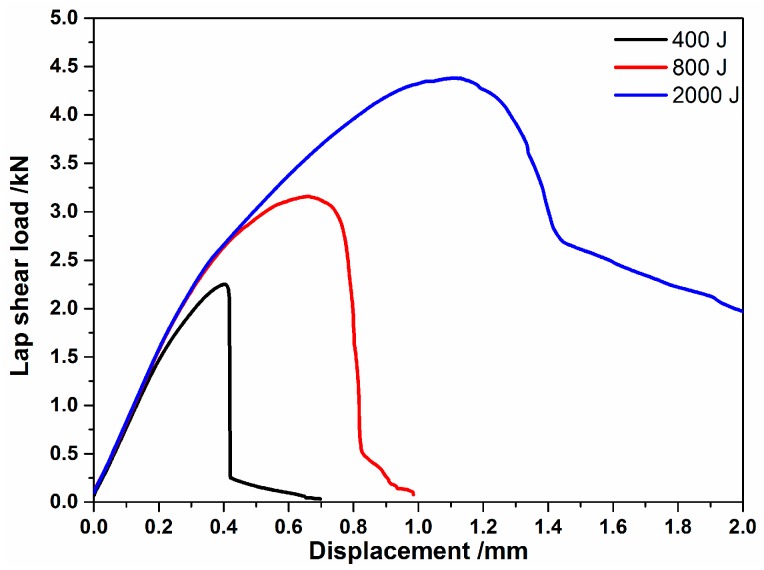
The lap shear load–displacement curves recorded for various welding energies.

Typical load-displacement curves of interfacial cleavage joints (produced by low weld energies of 400 J and 800 J) and a pull-out failure of joints (produced by a higher energy of 2000 J), are presented in Figure 9. It can be observed that, during the pull-out mode, the joints presented a good ductile behavior. However, during low welding energy conditions, the joints also displayed a ductile behavior before fracture.

**Figure 10:**
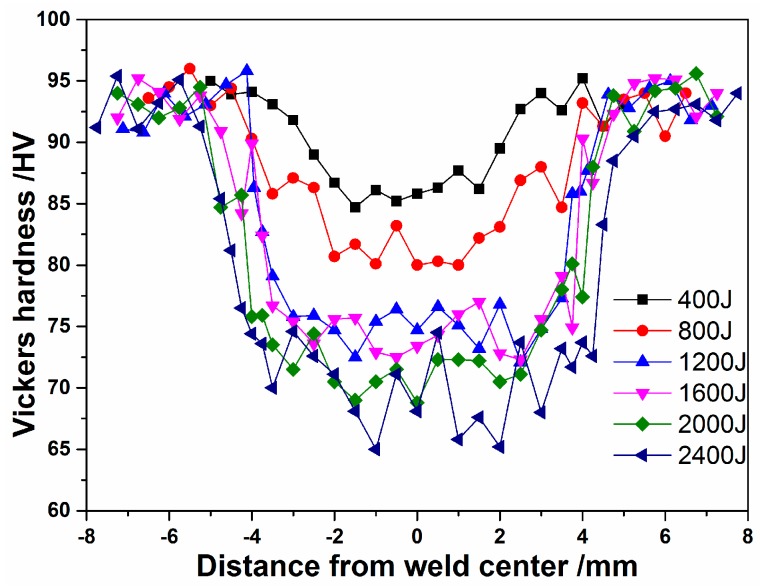
Hardness distributions on the transverse weld zone of the joints with various weld energy levels.

In Figure 10, the Vickers microhardness measurements along the transverse weld zone of the USW copper joints, under various welding energies, are presented. The hardness values of the bond zone decreased as the welding energy increased, being simultaneously lower than the corresponding values of the base metal. The hardness of the base metal was in the 90–95 Hv range, having been in accordance with the ½ H condition. The hardness of the bond zone, produced under a low welding energy, was slightly lower than the hardness of the base metal, in spite of the smaller grain size. It was suggested that the hardness of the weld zone was independent of the grain size. Therefore, other factors that affected the hardness value of the bond zone were produced during the USW, such as the annealing softening and the dislocation density. As it is well known, a high amount of heat was generated by friction and plastic deformation during USW, which leads to remarkable annealing softening. The heat can reduce the hardness of the bond zone. On the other hand, as reported by Lee et al. [25], the dislocation density of the weld zone was remarkably decreased when compared to the dislocation density of the base metal of the FSW copper. The lower dislocation density could be more accountable for the lower hardness of the stir zone than the harness of the base metal. Consequently, at low energy conditions of 400–800 J, although the grain size of the bond zone was obviously smaller than the grain size of the base metal, due to the annealing softening effect and low dislocation density being the dominant effects during the USW, the hardness values of the bond zone were still lower than the hardness values of the base metal. Furthermore, similar results were reported in FSW copper joints studies [25,26].

## 4. Conclusions

In the present work, an investigation of the microstructural and mechanical properties of Cu-Cu joints fabricated by USW was performed. The main conclusions deriving from this work are:

1. The copper sheets, of 0.8–0.8 mm in thickness, were successfully welded by high power ultrasonic welding. Defect-free joints were obtained from a wide welding energy range of 1600 to 2400 J, at a constant clamping force of 2350 N. The joints can be divided into four distinct regions, including the sonotrode imprint zone, the bond zone, the anvil imprints zone, and the base metal.
2. During low welding energy usage, weld formation is developed heterogeneously at the specific regions under the sonotrode tip ridges. The interfaces were straight. As the welding energy increased, the plastically deforming zone expanded, and the weld interface changed from sinusoidal, to a convoluted wavy pattern.
3. There was an increasing trend in the mean grain size of the weld zone, as the welding energy increased. The grain size of the bond zone was significantly smaller than the base metal grain size, when the welding energy was low. Dynamic recrystallization occurred during the USW of copper.
4. The load curves of the joints can be described in three stages: the initiation stage, the growth stage, and the plateau stage. The peak loads were mainly decided by the metallic joining area. Two representative fracture modes existed; the interfacial cleavage mode and the pull-out mode of ultrasonic weld joints.
5. The hardness values of the bond zone decreased as the welding energy increased, resulting in lower values than those of the base metal hardness.
